# Purpose in life as a predictor of capability well-being among older adults in Thailand: a cross-sectional study

**DOI:** 10.1186/s12877-026-07929-2

**Published:** 2026-07-17

**Authors:** Yuki Kumagai, Ishtiaq Ahmad, Chitose Shimizu, Yoshihisa Shirayama, Miyoko Okamoto, Shori Takaoka, Itsaranuwat Yongpisanphop, Motoyuki Yuasa

**Affiliations:** 1 Department of Global Health Research, Graduate School of Medicine, Juntendo University, Tokyo, Japan; 2 Faculty of International Liberal Arts, Juntendo University, Tokyo, Japan; 3 College of Public Health Science, Chulalongkorn University, Bangkok, Thailand; 4 Wat Phleng Hospital, Ratchaburi, Thailand

**Keywords:** Well-being, Older adults, Spiritual health, Purpose in life, Thailand

## Abstract

**Introduction:**

Contemporary discussions on the definition of health, as highlighted by the World Health Organization (WHO), emphasize the significant role of spiritual health (SH) in contributing to overall well-being, alongside physical, mental, and social dimensions. In Thailand, the rapidly ageing population has elevated the importance of improving the well-being of older adults, making it a critical national concern. In this context, SH plays a crucial role and is recognized in Thai national legislation as a key element of health. However, specific research on the relationship between SH and well-being among Thai older adults remains limited. This study aimed to investigate the current state of well-being among independent older adults in Central Thailand and to examine factors associated with SH and well-being.

**Methods:**

This cross-sectional study was conducted from June 1 to July 31, 2025, using a Paper-based questionnaire survey targeting 500 older adults aged 60 and over within Ratchaburi Province, central Thailand. The well-being was assessed using the ICECAP-O (ICEpop CAPability measure for Older people). Descriptive statistics (frequency, percentage) and univariate analysis (one-way ANOVA, t-test) were conducted. Multivariate linear regression analysis was performed to examine the relationships between well-being, socioeconomic characteristics, health perception, and the Purpose in Life (PIL) test. Stata/BE17.0 was used, with a significance level set at *p* < 0.05. This study was approved by the ethics review boards of both countries’ institutions: Juntendo University, Japan (E23-0364-M03), and Chulalongkorn University, Thailand (COA No.197/68).

**Results:**

Among the 471 valid responses, the median ICECAP-O tariff scores was 0.868, and participants were classified into low (10.6%), moderate (33.8%), and high (55.6%) PIL groups. In multivariate linear regression analysis, the adjusted predictive margins revealed a stepwise gradient, where ICECAP-O tariff scores for the moderate and high PIL groups were 0.098 and 0.149 points higher, respectively, compared to the low PIL group (0.735). Subjective health, economic status, and caregiving roles were also positive predictors, whereas older age was negatively associated.

**Conclusion:**

Given the critical role of spiritual health, targeted strategies to promote PIL are needed to enhance healthy aging. The findings highlight PIL as the strongest predictor of capability well-being, alongside subjective health and financial factors within the Thai cultural context.

**Supplementary Information:**

The online version contains supplementary material available at https://doi.org/10.1186/s12877-026-07929-2.

## Introduction

Population aging is a defining global phenomenon of the 21st century. According to the United Nations (UN), the number of older adults aged 60 years or over is projected to double by 2050 [1, 2]. By 2030, 1 in 6 people in the world will be aged 60 years or over. The World Health Organization (WHO) projects that the proportion of the global population aged 60 years and older will nearly double from 12% to 22% between 2015 and 2050 [3].

Thailand is currently experiencing a dramatic demographic transition characterized by a rapid increase in the older population [4]. Recent public health data indicate that the number of older adults aged 60 years and over has reached approximately 14 million, constituting over 20% of the total population. This signifies that Thailand has officially entered a “complete aged society” [5]. This shift is primarily driven by a drastic decline in the total fertility rate (TFR), which has fallen to approximately 1.0 to 1.1, a level significantly below the population replacement rate [6]. The WHO South-East Asia Region forecasts that the elderly population will comprise 36% of the total by the 2050s, signifying a demographic shift toward a “super-aged society” [4]. This demographic shift poses significant challenges to the healthcare system and necessitates a comprehensive approach to maintaining the quality of life for older adults.

Traditionally, health has been defined in three dimensions: physical, mental, and social well-being. However, there is a growing global consensus regarding the inclusion of a fourth dimension as spiritual health (SH) [7]. The WHO has long discussed the integration of the spiritual dimension into the definition of health to ensure a holistic approach to human well-being. SH often conceptualized through constructs such as meaning and purpose in life, is increasingly recognized as a vital determinant of overall well-being, distinct from religious adherence [8].

In alignment with global trends, the Thai government has prioritized the promotion of holistic well-being in its national health policies. Uniquely, Thailand’s National Health Act B.E. 2550 (2007) explicitly defines health as consisting of four dimensions: physical, mental, social, and spiritual well-being [9]. Within this framework, SH is regarded as a fundamental element for achieving happiness and societal harmony. Consequently, fostering SH has become a key strategic objective in Thailand’s efforts to support its aging population.

Despite increasing policy emphasis, empirical evidence regarding the SH of Thai older adults remains limited. Although its conceptual importance is well-recognized, quantitative data characterizing the prevalence and levels of SH in this demographic are scarce [5]. Furthermore, the mechanisms through which SH, specifically its measurable core component, the Purpose in Life (PIL), is associated with the well-being of the rapidly aging population remain insufficiently clarified [10]. Consequently, further investigation into the predictors of healthy life expectancy and the specific role of PIL is imperative to develop evidence-based strategies that promote inclusive well-being among Thailand’s aging population.

To address these gaps, this study employs the ICECAP-O to conceptualize the measure of overall well-being and five attributes of capability (Attachment, Security, Role, Enjoyment and Control). Unlike traditional measures focused on “functioning” or current health states, the capability approach emphasizes an individual’s opportunity to “do and be” what they value [11]. The attributes are fundamentally linked to various sociodemographic and spiritual factors. Specifically, Attachment is formed by social and familial networks, such as co-residence with children or participation in elderly clubs. Security relates to financial stability and health-related certainty, influenced by factors like pension receipt and household debt. The role covers the ability to feel valued through social contributions, including volunteering or caregiving. Enjoyment reflects opportunities for leisure and spiritual fulfilment, while Control indicates the autonomy to make independent life choices, often constrained by physical mobility or economic dependency. This framework is particularly suited for assessing SH, as it accommodates diverse, value-based interpretations of a fulfilling life.

Building on this background, the present study seeks to answer the following research question. How PIL an indicator of SH associated with well-being among independent older adults in Thailand? We hypothesize that SH, measured by a strong sense of PIL, serves as a significant positive predictor of well-being. Accordingly, this study aims to investigate the current status of well-being among independent older adults in Central Thailand and examine factors associated with SH and well-being. In this study, while SH is recognized as a broad theoretical dimension, we specifically focus on PIL to operationally evaluate this concept.

## Methodology

### Study setting and participants

Paper-based questionnaires were distributed at local older adults’ community club, volunteer organizations, and health centers. Community leaders were previously informed about the inclusion criteria (60 years or older, living in the community, and capable of responding independently) and helped to invite suitable participants. Written informed consent was obtained from all respondents prior to completing the questionnaire.

**Fig. 1:**
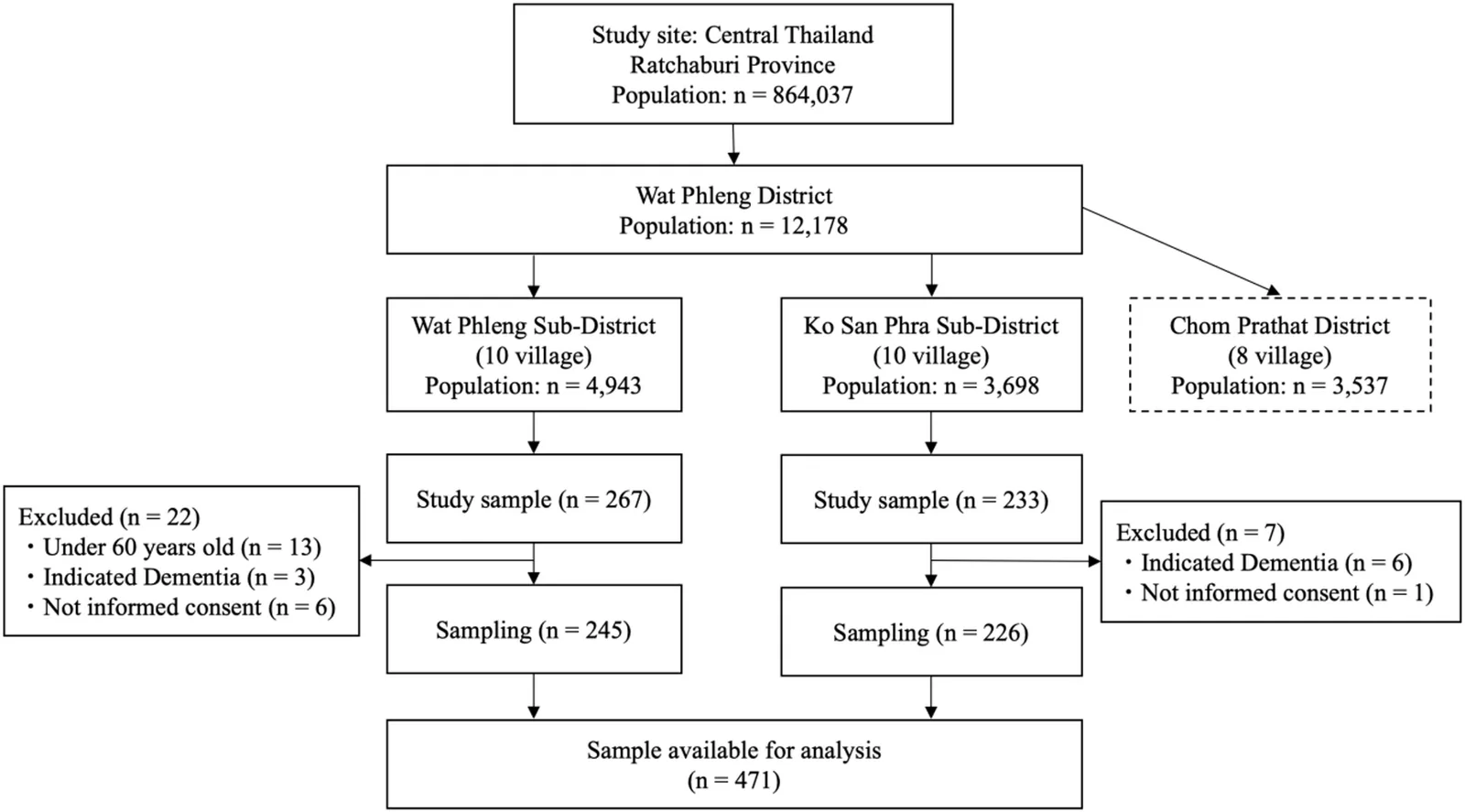
Flowchart of participant enrollment

A total of 500 responses were collected through this multistage purposive sampling technique (Fig. 1). This approach was employed to select participants from this region, which is characterized by its significant economic, political, and cultural integration with Bangkok [12]. Ratchaburi Province was specifically selected due to its representative demographic structure, with a focus on Wat Phleng District, which reports the highest proportion of older residents (34%) in the province [13]. Among the three sub-districts within Wat Phleng, Chom Prathat was excluded due to the high number of unregistered older residents. Consequently, the participants were recruited from the Ko San Phra and Wat Phleng sub-districts.

### Sample size calculation

The required sample size was determined using Taro Yamane’s formula [14], calculated as follows:

$$\:n=\frac{N}{1+N{e}^{2}}$$

Where N represents the total population of older adults in Thailand (*N* = 14,027,411) [15], and represents the margin of error (e = 0.05 for a 95% confidence level). The calculation yielded a minimum required sample size of approximately 400. We received 500 responses. After excluding participants who did not fit the inclusion criteria (*n* = 29), 471 valid responses were retained for the final analysis. 63 cases with missing data were handled using Multiple Imputation by Chained Equations (MICE) with 20 imputed datasets, and results were pooled using Rubin’s rules. (the detailed of multiple imputation procedures are described in Supplementary Information 1) This final sample size exceeds the initial requirement, thereby ensuring sufficient statistical power for the study’s objectives.

### Eligibility criteria

Participants in this study were community-dwelling older adults aged 60 years and older residing in Ratchaburi Province. To be eligible, individuals were required to be active members of an elderly club, native Thai speakers, and capable of living independently with stable general health. Both verbal and written informed consent were obtained from all participants prior to their inclusion. Conversely, individuals diagnosed with dementia were excluded from the study. Exclusion also applied to potential participants identified by public health officers or local village health volunteers as having cognitive impairment, an inability to live independently, or unstable general health conditions.

### Study instruments

#### Dependent variables

The dependent variable in our study is well-being, which was measured using the ICEpop CAPability measure for older people (ICECAP-O). It was developed based on Sen’s capability approach; this preference-based instrument captures broad aspects of quality of life beyond health status that are essential for older adults [16]. The scale comprises five attributes: (1) Attachment (love and friendship), (2) Security (thinking about the future without concern), (3) Role (activities that provide a sense of value), (4) Enjoyment (enjoyment and pleasure), and (5) Control (independence). Each attribute is rated on a four-level scale, ranging from full capability (level 4) to no capability (level 1). Index scores were calculated using the UK population-based tariffs established by Coast et al. [17], which utilizes Best-Worst Scaling (BWS) to reflect the preferences of the general older population. The scoring follows an additive model in which coefficients for each selected level are summed. The attributes are weighted unequally, with Attachment carrying the highest weight (coefficient of 0.2535 for the top level), followed by Control (0.2094), Role (0.1923), Security (0.1788), and Enjoyment (0.1660) [17]. Details on the preparation and processing of ICECAP-O tariff scores are provided in Supplementary Information 1. The resulting index score ranges from 0.0 (no capability) to 1.0 (full capability) and was treated as a continuous variable in the analysis [11]. As an official Thai version of the ICECAP-O was unavailable, a translation was conducted following the guidelines provided by the Bristol Medical School, University of Bristol. This process involved forward and backward translation by two independent experts. A pilot study was subsequently conducted with 33 older adults in a community in Ratchaburi with similar characteristics to the target population. While the pilot results showed a significant association between the ICECAP-O and SH (*p* = 0.013), the initial internal consistency was moderate (Cronbach’s α = 0.66). Consequently, the Thai version was revised based on recommendations from the ethics review committee, which reconfirmed its face and content validity. The Cronbach alpha value of this instrument in our main survey data was 0.87.

#### Independent variables

The independent variable in our study is SH, measured by Part A of the PIL test [18]. We consider this instrument particularly appropriate for assessing SH in the Thai older adult population, as it focuses on an existential sense of meaning and the “will to meaning” rather than specific religious doctrines, making it culturally adaptable and suitable for diverse backgrounds. This scale was developed based on Viktor Frankl’s concept of the existential vacuum and logotherapy, positing that the search for meaning is a primary human motivation [19]. The PIL is a 20-item instrument; responses for each item are recorded on a 7-point Likert scale ranging from 1 (Strongly Disagree) to 7 (Strongly Agree). The variable is constructed by summing the scores of each item, yielding a total score ranging from 20 to 140. Following established classification criteria, scores ranging from 20 to 91 were categorized as low PIL, 92 to 112 as moderate PIL, and 113 to 140 as high PIL [20]. The original PIL test was written in English, and since then, it has been translated into many languages [21]. We included authentic Thai translations of this instrument in our questionnaire, as used by a previous study (Cronbach’s α = 0.84) [22]. The Cronbach alpha value of this instrument in our survey data was 0.96.

Data on other independent variables, such as sociodemographic and economic factors, social engagement, and health-related factors were collected using the following variables: Sociodemographic factors included age, gender, education level, marital status, having children, and co-resident’s status. Economic factors included employment status, longest job, income level, economic status, household debt, and pension receipt. Health-related and social engagement factors included current illness, caregiving within the household, subjective health perception, social activities, and volunteering. Additionally, religious background specifically included faith status and religion type. The validated questionnaire CECAP-O and PIL in the Thai language was used in this study. The English version of the questionnaire is provided as a supplementary file (Supplementary Information 2). The questionnaire was organized into three separate parts, (i) sociodemographic, economic factors, social engagement and health-related items (independent variables), (ii) ICECAP-O (dependent variable) and (iii) the PIL test (independent variable). This procedural separation was used to reduce the potential bias of the common method.

### Statistical analysis

Descriptive statistics summarized respondent characteristics using frequencies and percentages for categorical variables, and means with standard deviations (SD) or medians with interquartile ranges (IQR) for continuous variables. Following the OECD Guidelines on Measuring Subjective Well-being [23], we prioritized reporting central tendencies that are robust against the negative skewness and ceiling effects typically observed in well-being data. To explore differences across covariates, ICECAP-O tariff scores were treated as a continuous variable to preserve the full informational value of the capability measure, with group differences evaluated using Student’s t-tests and one-way analysis of variance (ANOVA), as appropriate. Univariate linear regression analyses were performed to identify unadjusted associations between capability well-being and individual independent variables. Variables found to be statistically significant in univariate analyses (*p* < 0.20), along with theoretically essential covariates such as age and gender, were included in multivariable linear regression models (ordinary least squares; OLS). Furthermore, the assumptions of the OLS regression were checked. The normality of residuals was visually confirmed using Q-Q plots, and homoscedasticity was verified via residual versus fitted plots and the Breusch-Pagan test, indicating no significant deviations from these assumptions. Regarding economic factors, subjective economic status was prioritized as the representative variable to mitigate potential multicollinearity among income, household debt status, pension, and employment status. The variable was the largest effect size (η^2^ = 0.149) in the univariate analysis among economic variables. This selection reflects perceived financial security as a direct determinant of individual well-being. Although both social activities and volunteering showed significant associations in the univariate analysis regarding social engagement, social activities demonstrated a larger effect size (Cohen’s d = 0.393 vs. 0.313). Therefore, to avoid multicollinearity, social activities were selected for the final model to represent social engagement. Other non-significant variables in the univariate analysis were excluded to ensure model parsimony and prevent overfitting. Details regarding the justification for the analytical approach are provided in Supplementary Information 3. To illustrate practical significance, adjusted predictive margins were estimated to visualize the specific impact of PIL on capability well-being. Multicollinearity was further evaluated using variance inflation factors (VIF), with values < 5 indicating no significant multicollinearity. Statistical significance was set at *p* < 0.05, and 95% confidence intervals were reported. All Statistical analyses were performed using Stata version 17 (Stata Corp LLC, College Station, TX, USA).

### Ethical review

This study was approved by the ethics review boards of both institutions: Juntendo University, Japan (E23-0364-M03) and Chulalongkorn University, Thailand (COA No.197/68). Written informed consent was obtained from all participants prior to data collection. To ensure confidentiality, all responses were anonymized and securely stored in a password-protected offline system.

## Results

A total of 471 participants were included in the final analysis. The average age was 69.17 years (SD = 7.55), with a range of 60 to 97 years. As summarized in Table 1, the majority of the participants were women (60.9%), aged 60–69 years (59.2%), primary or lower secondary school graduates (69.0%), married or cohabiting (54.6%), had children (83.0%), lived with others (90.9%), and identified as Buddhist (84.7%). Regarding economic status, participants reported having no financial difficulty (46.9%), household debt (61.4%), and a monthly income below 20,000 baht (51.0%). Most were currently working (66.0%), with self-employment (29.7%) being the most common longest-held occupation. Regarding health status, the participants reported a current illness (69.6%). Subjective health perception was predominantly positive, including “good” or “very good” (59.2%) versus “poor” (2.8%). Social engagement included activity participation (59.9%) and volunteering (41.6%), and a substantial proportion (62.0%) was involved in household caregiving.

**Table 1:** Sociodemographic characteristics of study participants (*n* = 471)

Socioeconomic Characteristics	*n* = 471	Percent (%)
Gender (*n* = 470*)		
Male	183	38.9
Female	287	60.9
Age		
60–69	279	59.2
70–79	134	28.5
80+	58	12.3
Education Level		
Primary or lower secondary	325	69.0
Upper secondary education / Higher Vocational	90	19.1
Bachelor’s or Higher	56	11.9
Marital Status		
Single (never married)	69	14.7
Married / Cohabiting	257	54.6
Divorced / Living Separately	47	10.0
Widowed	98	20.8
Children		
Has children	391	83.0
No children	80	17.0
Co-residency Status		
Lives alone	43	9.1
Lives with others	428	90.9
Current Illness		
No illness	143	30.4
Has illness	328	69.6
Employment Status		
Worker	311	66.0
Not working	160	34.0
Longest Job		
Self-employed	140	29.7
Others	331	70.3
Economic Status		
No difficulty	221	46.9
Somewhat manageable	120	25.5
Slightly worried	98	20.8
Very worried	32	6.8
Household Debt Status		
Has debt	289	61.4
No debt at all	182	38.6
Income Level		
< 20,000 baht	240	51.0
20,000–39,999 baht	151	32.1
40,000 + baht	80	17.0
Pension Receipt Status		
No	399	84.7
Yes	72	15.3
Caregiving within the household		
No	179	38.0
Yes	292	62.0
Belief or Faith Status		
Have and depend on a lot	163	34.6
Have and depend on somewhat	271	57.5
Do not have or Not sure	37	7.9
Religion Type		
Buddhism	399	84.7
Christianity (Protestant)	4	0.9
Christianity (Roman Catholic)	56	11.9
Animism / Folk beliefs	2	0.4
None	10	2.1
Social Activities		
Does not participate	189	40.1
Participates	282	59.9
Volunteering		
Does not participate	275	58.4
Participates	196	41.6
Subjective Health Perception		
Very good health	41	8.7
Good health	238	50.5
Not very good health	179	38.0
Poor health	13	2.8

* Gender diverse individuals were excluded because there was only one person

**Fig. 2:**
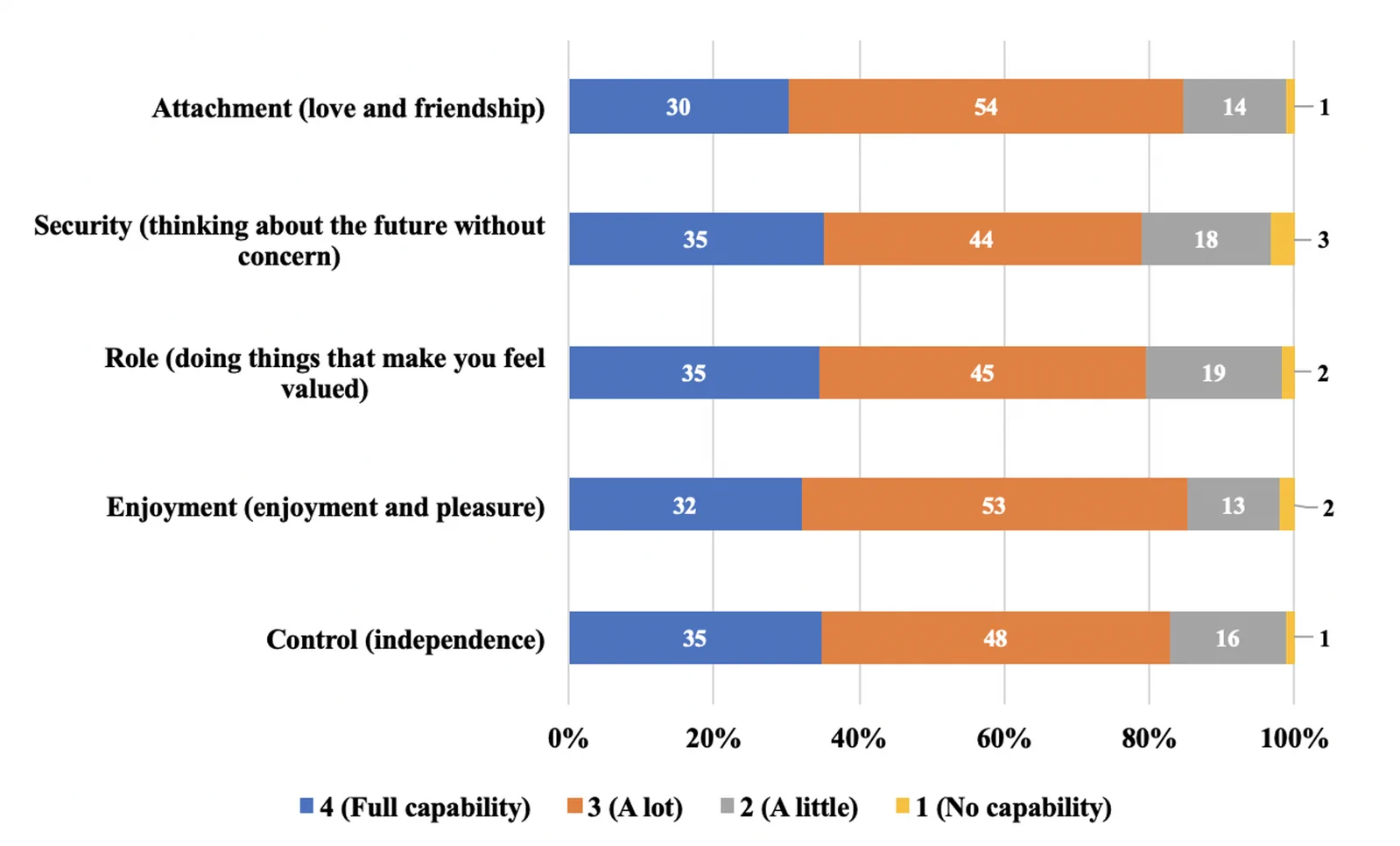
Capability level responses across ICECAP-O attributes

**Fig. 3:**
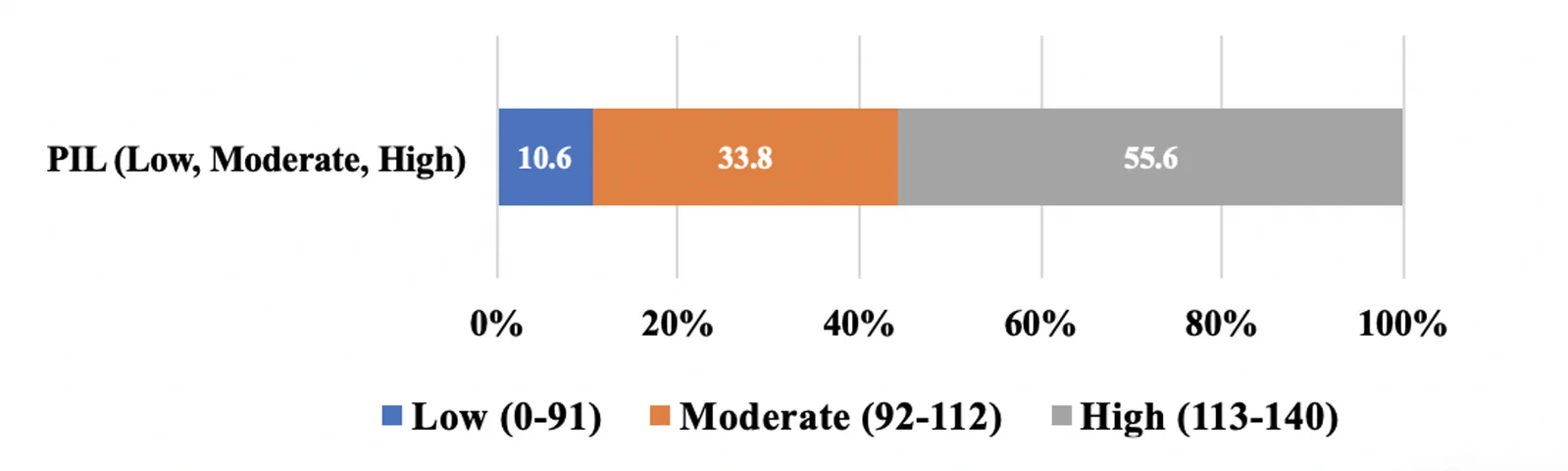
Distribution of PIL score categories among study participants

The mean ICECAP-O tariff scores was 0.86 (SD = 0.13), with a median of 0.87 (range: 0.15–1.00) (Table 2). As illustrated in Fig. 2, high capability levels (reporting “full” or “a lot”) exceeded 80% in the Attachment, Enjoyment, and Control domains, while reports of “no capability” remained minimal (1%–3%) across all attributes. SH, measured by the PIL test, showed a mean total score of 112.42 (SD = 19.69), ranging from 26 to 140 (Table 2). Based on established cutoffs in Figs. 3 and 55.6% of participants were categorized as having “high” PIL (scores 113–140), 33.8% as “moderate” PIL (scores 92–112), and 10.6% as “low” PIL (scores 20–91).

**Table 2:** Descriptive statistics of age, ICECAP-O tariff scores, and PIL total scores

Domain	Mean	Median	SD	25%	75%	Minimum	Maximum
Age	69.17	68	7.551	63	73	60	97
Well-being	0.861	0.868	0.131	0.848	0.873	0.147	1.000
(ICECAP-O tariff scores)							
Spiritual Health	112.42	115	19.69	110.52	114.31	26	140
(PIL total scores)							

### Factors associated with well-being (univariate analysis)

A univariate analysis was conducted to examine the unadjusted associations between modifying factors and well-being, with the ICECAP-O tariff scores treated as a continuous variable (Table 3). The analysis revealed that age and education level were significantly associated with capability well-being (*p* < 0.001). The mean ICECAP-O tariff scores was 0.874 in the 60–69 age group and 0.751 in the 80 + age group. Regarding education level, participants with a bachelor’s degree or higher had a mean score of 0.908. Compared to this group, those with primary education or lower had a mean score of 0.833 (*p* < 0.001). Marital status was also significantly associated with capability well-being (*p* = 0.029). No statistically significant differences were observed for gender, presence of children, or co-residency status.

**Table 3:** Univariate analysis of ICECAP-O tariff scores by participant characteristics

Modifying Factors	Well-being (ICECAP-O tariff scores)			
	Mean	*N*	*p* value	Effect size*
Gender (*n* = 470)			0.602	0.049
Male	0.855	183		
Female	0.848	287		
Age			< 0.001	0.074
60–69	0.874	279		
70–79	0.846	134		
80+	0.751	58		
Education Level			< 0.001	0.038
Primary or lower secondary	0.833	325		
Upper secondary education / Higher Vocational	0.881	90		
Bachelor’s or Higher	0.908	56		
Marital Status			0.029	0.019
Single (never married)	0.869	69		
Married / Cohabiting	0.862	257		
Divorced / Living Separately	0.806	47		
Widowed	0.831	98		
Children			0.597	0.065
Has children	0.849	391		
No children	0.859	80		
Co-residency Status			0.544	0.097
Lives alone	0.864	43		
Lives with others	0.850	428		
Current Illness			0.010	0.257
No illness	0.877	143		
Has illness	0.840	328		
Employment Status			< 0.001	0.481
Worker	0.874	311		
Not working	0.806	160		
Longest Job			0.379	0.088
Self-employed	0.860	140		
Others	0.847	331		
Economic Status			< 0.001	0.149
No difficulty	0.890	221		
Somewhat manageable	0.849	120		
Slightly worried	0.822	98		
Very worried	0.671	32		
Household Debt Status			0.123	0.146
Has debt	0.843	289		
No debt at all	0.864	182		
Income Level			< 0.001	0.048
< 20,000 baht	0.822	240		
20,000–39,999 baht	0.892	151		
40,000 + baht	0.860	80		
Pension Receipt Status			< 0.001	0.475
No	0.840	399		
Yes	0.908	72		
Living with someone who requires your caregiving			0.001	0.315
No	0.823	179		
Yes	0.868	292		
Belief or Faith Status			< 0.001	0.059
Have and depend on a lot	0.886	163		
Have and depend on somewhat	0.843	271		
Do not have or Not sure	0.751	37		
Religion Type			0.118	0.016
Buddhism	0.851	399		
Christianity (Protestant)	0.955	4		
Christianity (Roman Catholic)	0.856	56		
Animism / Folk beliefs	0.655	2		
None	0.795	10		
Social Activities			< 0.001	0.393
Does not participate	0.817	189		
Participates	0.873	282		
Volunteering			0.001	0.313
Does not participate	0.832	275		
Participates	0.877	196		
Subjective Health Perception			< 0.001	0.226
Very good health	0.958	41		
Good health	0.882	238		
Not very good health	0.807	179		
Poor health	0.551	13		
PIL			< 0.001	0.235
Low	0.678	50		
Moderate	0.822	159		
High	0.901	262		

*Continuous outcomes (ICECAP-O tariffs) were compared using the t-test (two groups) andOne-way ANOVA (≧ 3 groups). t-test (Effect size: Cohen’s d = 0.20 Small, 0.50 Medium, over 0.80 Large), One-way ANOVA (Effect size: Eta-squared η= 0.01 Small, 0.06 Medium, over 0.14 Large)

Subjective economic status showed a different gradient. Compared to participants reporting “no difficulty” (Mean = 0.890), those who were “very worried” about their financial situation had a significantly lower mean score of 0.671 (*p* < 0.001). This suggests that perceived financial security is significantly associated with the capability of well-being. Higher income levels and receipt of a pension were also linked to significantly higher mean scores (*p* < 0.001). Household debt status showed no significant difference (*p* = 0.123). Health and social engagement factors were strongly linked to the capability for well-being. Participants with no current illness reported a higher mean score than those with an illness (0.877 vs. 0.840, *p* = 0.010). Regarding social engagement, individuals involved in social activities (*p* < 0.001) and volunteering (*p* = 0.001) exhibited significantly higher mean scores.

Subjective health perception showed an affected association (*p* < 0.001). Mean score ranged from 0.958 for those in “very good health” to 0.551 for those in “poor health.” Notably, assuming a caregiving role was associated with higher capability well-being. Caregivers reported a higher mean score compared to non-caregivers (0.868 vs. 0.823, *p* = 0.001). Faith or Belief dependency was also significantly associated with well-being (*p* < 0.001).

SH, measured by the PIL test, demonstrated a strong association with capability well-being (*p* < 0.001). A distinct gradient was observed across groups. The mean ICECAP-O tariff scores was 0.901 for the high PIL group, 0.822 for the moderate PIL group, and 0.678 for the low PIL group.

### Factors associated with capability well-being (multivariable linear regression analysis)

A multivariable linear regression analysis was conducted to identify factors independently associated with capability well-being (ICECAP-O) (Table 4). The analysis revealed that PIL had the strongest independent association with well-being compared to participants with low PIL. Those with “Moderate” (Coefficient = 0.098, *p* < 0.001) and “High” (Coefficient = 0.149, *p* < 0.001) levels exhibited significantly higher ICECAP-O tariff scores, indicating a significant linear trend across increasing PIL levels. Subjective health perception also showed a strong, significant association; participants rating their health as “poor” had substantially lower well-being score (Coefficient = − 0.269, *p* < 0.001) compared to those with “very good” health. Similarly, economic status was a significant predictor, with participants expressing “very worried” about their finances showing a lower score (Coefficient = − 0.128, *p* < 0.001) compared to those with no financial difficulties. And, caregiving was positively associated with well-being; those taking care of others had significantly higher scores (Coefficient = 0.046, *p* < 0.001) than non-caregivers. In contrast, Age (80 years and older) was associated with lower capability well-being (Coefficient = − 0.061, *p* < 0.001). Gender, Education level, Marital Status, Current illness, Faith, Religion Type, and Social Activities were not statistically significant in the adjusted model.

**Table 4:** Multivariable linear regression analysis of factors associated with ICECAP-O tariff scores

Variable	Coefficient	95% CI	*P* value
Gender (base: male)			
Female	0.007	-0.014, 0.029	0.521
Age (base: 60–69)			
70–79	0.002	-0.021, 0.025	0.872
80+	-0.061	-0.094, -0.028	< 0.001
Education (base: Primary or lower secondary)			
Upper secondary education / Higher Vocational	0.011	-0.016, 0.038	0.419
Bachelor’s or Higher	0.012	-0.022, 0.045	0.498
Marital Status (base: Single, never married)			
Married / Cohabiting	-0.010	-0.040, 0.020	0.532
Divorced / Living Separately	-0.041	-0.082, 0.000	0.052
Widowed	-0.009	-0.045, 0.026	0.598
Current Illness (base: No illness)			
Has illness	0.017	-0.007, 0.041	0.164
Economic Status (base: No difficulty)			
Somewhat manageable	-0.021	-0.046, 0.005	0.114
Slightly worried	-0.037	-0.065, -0.009	0.010
Very worried	-0.128	-0.172, -0.083	< 0.001
Living with someone who requires your caregiving			
Yes	0.046	0.025, 0.067	< 0.001
Faith (base: Have and depend on a lot)			
Have and depend on somewhat	-0.006	-0.028, 0.017	0.624
Do not have or Not sure	-0.008	-0.058, 0.042	0.763
Religion Type (base: Buddhism)			
Christianity (Protestant)	0.047	-0.062, 0.155	0.399
Christianity (Roman Catholic)	-0.006	-0.037, 0.025	0.690
Animism / Folk beliefs	-0.329	-0.548, -0.110	0.003
None	-0.031	-0.112, 0.050	0.453
Social Activities (base: Does not participate)			
Participates	0.008	-0.014, 0.030	0.459
Subjective Health Perception (base: Very good health)			
Good health	-0.068	-0.106, -0.031	< 0.001
Not very good health	-0.111	-0.152, -0.069	< 0.001
Poor health	-0.269	-0.344, -0.193	< 0.001
PIL (base: Low)			
Moderate	0.098	0.061, 0.135	< 0.001
High	0.149	0.111, 0.187	< 0.001

### Adjusted predictive margins of capability by PIL (Table 5; Fig. 4)

**Table 5:** Adjusted predictive margins of ICECAP-O tariff scores by levels of PIL

Purpose in Life (PIL)	Adjusted Margin*	95% CI	*P* value
Low	0.735	0.701 to 0.768	< 0.001
Moderate	0.833	0.815 to 0.850	< 0.001
High	0.884	0.870 to 0.898	< 0.001

*CI  *Confidence Interval

* Values represent predictive margins adjusted for all other covariates in the multiple linear regression model

**Fig. 4:**
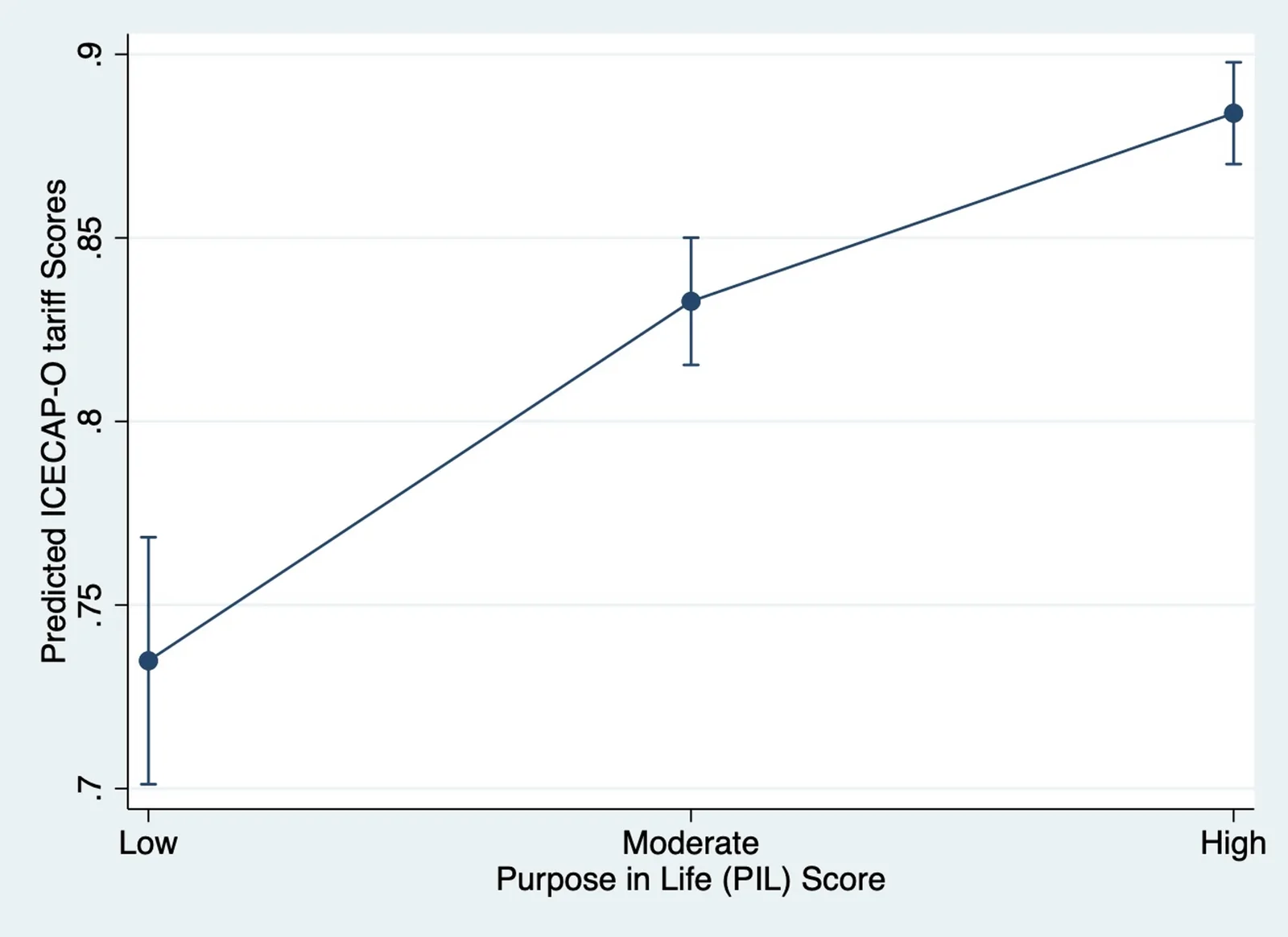
Relationship between PIL score and ICECAP-O tariff scores

To further quantify the independent relationship between PIL and the capability of well-being, we calculated the predictive margins of the ICECAP-O tariff scores adjusted for all covariates in the multivariable model. The adjusted mean ICECAP-O tariff scores was the lowest for participants in the Low PIL group at 0.735 (95% CI: 0.701–0.768). The score significantly increased to 0.833 (95% CI: 0.815–0.850) for the Moderate PIL group and reached the highest level of 0.884 (95% CI: 0.870–0.898) for the High PIL group. The distinct, not overlapping confidence intervals between the Low PIL group and the other groups confirm a statistically significant, stepwise gradient in capability scores associated with higher levels of PIL.

## Discussion

This study investigated the status of ICECAP-O among independent older adults in Central Thailand and examined its association with SH. The results demonstrated that the participants maintained a generally high level of well-being and SH. The most significant finding was that PIL served as the strongest independent predictor of ICECAP-O tariff scores in the multivariable analysis (Coefficient = 0.149, 95% CI: 0.111 to 0.187), exhibiting a clear stepwise gradient according to the level of PIL (Table 4; Fig. 4). This result strongly supports our hypothesis that SH is a vital element for maintaining well-being in older adults. Its magnitude of association surpassed both economic and health factors. In this study, the mean ICECAP-O tariff scores was 0.861 (SD 0.131). This score is comparable to or even slightly higher than the average score reported in the general older population of the United Kingdom (0.832) [17]. The UK is a high-income country with an advanced welfare system. Therefore, it is noteworthy that Thai older adults in a developing region achieved such high capability scores. This suggests that factors other than economic wealth play a fundamental role in maintaining quality of life. Specifically, social and cultural capital appear to be key drivers in this population.

Over 80% of respondents reported the top two levels of capability (“Full capability” or “A lot”) in the “Attachment (84%)”, “Enjoyment (85%)” and “Control (83%)” domains (Fig. 2). This finding strongly reflects the cultural fabric of Thai society. Thai culture is characterized by strong intergenerational solidarity and community cohesiveness. In Thailand, older adults typically live with or near their children and are deeply integrated into village life [24]. Previous qualitative research has highlighted that “connectedness” with family and neighbors is the primary source of psychological well-being for Thai older adults [25], our study results to consistent these studies. Moreover, the Village Health Volunteer (VHV) system plays a crucial role in supporting this “Security” and “Attachment”. In Thailand, over one million VHVs provide basic health monitoring and social support to older adults in their communities [26]. The volunteers visit the older adults regularly. They check their health status, deliver essential medications and caregiving. This robust community-based network likely provides older adults with a profound sense of safety and control. It ensures them that they will not be abandoned even if their health declines. Consequently, this may contribute to the high scores (79%) in the “Security” domain of the ICECAP-O tariff scores.

SH, the mean PIL score was 112.42 (Table 2), 55.6% of participants were classified into the “High” PIL group (Fig. 3). This score is comparable to or higher than that reported in Japan (42.9%) [27], suggesting that cultural and health systems are fundamentally associated in Thailand. This high prevalence of strong life purpose is deeply rooted in the Thai cultural concept of “Tham Boon” (merit-making). Importantly, in Thai society, “Tham Boon” is not limited to religious rituals at temples. It encompasses a broader spirit of mutual aid and altruism in daily life. For instance, helping neighbors and caring for family members are also considered good acts of accumulating merit [28]. This cultural belief system reframes “caregiving” and “community service” not as burdens, but as spiritual opportunities. By supporting others in the community or caring for grandchildren, older adults feel they are actively accumulating merit for their future lives. This perspective transforms their social roles into sources of spiritual meaning. Consequently, the act of “living for others” becomes a powerful engine for self-worth [29]. This likely explains why Thai older adults maintain high SH and a strong sense of purpose, even as they age.

The predicted ICECAP-O tariff scores rose significantly from 0.735 in the Low PIL group to 0.833 in the Moderate group and 0.884 in the High PIL group (Table 5; Fig. 4). The difference of nearly 0.15 points between the Low and High PIL groups is substantial. This magnitude exceeds the change typically associated with major physical morbidities. This implies that the lack of PIL may have a stronger negative association with capability than physical illness itself. The strong link between PIL and capability can be theoretically explained through Activity Theory and the concept of Successful Aging [30]. Activity Theory posits that maintaining social roles and engagement is fundamental to life satisfaction [31]. A high sense of PIL likely acts as a motivational engine. It encourages older adults to remain socially active, participate in community events, and engage in meaningful hobbies [32]. This motivation persists despite the physical limitations of aging. Consequently, these activities are likely reflected in higher scores in the “Role (80%)” and “Enjoyment (85%)” domains of the ICECAP-O tariff scores. Furthermore, it is important to acknowledge a potential conceptual overlap between PIL and the ICECAP-O. Both instruments capture deeply psychological and evaluative dimensions of human success, such as feeling valued and having a sense of control over one’s life. This inherent theoretical overlap likely explains a portion of the strong statistical association observed in our study. Consequently, rather than a mere methodological artifact, this thoughtful connection emphasizes the importance of the discussion by the WHO regarding the inclusion of a fourth dimension as SH of overall well-being.

Moreover, from the perspective of Resilience, PIL functions as a critical psychological buffer. Older adults inevitably face declines in physical health or the loss of loved ones. In such times, a strong sense of PIL provides the mental resilience needed to adapt. It allows them to focus on “what they can still do” (capability) rather than “what they have lost” (functioning) [33, 34]. This finding is consistent with recent meta-analyses showing that purposeful living significantly reduces the risk of functional disability and mortality [35]. In the context of Thailand’s rapid aging, promoting SH is not merely a philosophical pursuit. Instead, it is a practical public health intervention to extend healthy life expectancy and capability.

This study found subjective economic status, subjective health, and caregiving roles as significant determinants of capability well-being. Regarding economic factors, subjective economic status demonstrated a stronger association with well-being than objective income. In our analysis, compared to those with “No difficulty,” participants who were “Very worried” about their finances had a significantly lower score (Coefficient = -0.128, 95% CI: -0.172 to -0.083) with *p* < 0.001 (Table 4). This supports previous gerontological research suggesting that perceived financial adequacy is a more accurate predictor of psychological well-being than actual wealth [36]. For older adults, the “feeling of security” that they can manage future expenses is often more important than the absolute amount of money they possess. This finding directly aligns with the “Security” attribute of the ICECAP-O measure. It suggests that social safety nets, which reduce financial anxiety may be as effective as direct cash transfers in improving quality of life.

Subjective health was also a powerful predictor. Participants who perceived their health as “Poor” had markedly lower capability score (Coefficient = -0.269, 95% CI: -0.344 to -0.193) with *p* < 0.001 compared to those in “Very good” health. This is consistent with established literature indicating that self-rated health captures subtle bodily changes and psychological reserves that medical diagnoses might miss [37].

Notably, participants engaging in caregiving roles exhibited significantly higher capability scores (Coefficient = 0.046, 95% CI: 0.025 to 0.067) with *p* < 0.001 than non-caregivers (Table 4). This result challenges the common “caregiver burden” narrative. Instead, it supports the “Positive Aspects of Caregiving (PAC)” hypothesis [38]. Recent large-scale studies have indicated that caregivers often experience lower mortality and higher self-esteem due to the sense of being needed [39]. In the context of Thailand, this can be interpreted through the cultural norm of “Bun Khun” (gratitude and reciprocity). Caring for family members is viewed as a way to repay debts of gratitude and accumulate merit (Tham Boon). Therefore, caregiving provides older adults with a meaningful “Role” and enhances their social “Attachment”. It transforms a potential burden into a source of spiritual satisfaction and capability [40].

This study has several strengths. First, it is one of the few studies in Southeast Asia to assess older adults’ well-being using the ICECAP-O measure. Unlike traditional health-related quality of life instruments (e.g., EQ-5D), which focus primarily on physical and psychological functioning, the ICECAP-O is grounded in Amartya Sen’s capability approach [41]. This allowed us to capture a broader spectrum of well-being. Specifically, we evaluated what older adults “can do” and “be,” rather than merely the absence of disease. This approach aligns with the WHO’s modern definition of Healthy Ageing, which emphasizes functional capability over mere health status [42]. Second, this study provides novel empirical evidence linking SH (PIL) directly to capability well-being. Previous research has largely focused on the impact of spirituality on mental health outcomes, such as depression or anxiety [43, 44]. In contrast, our study highlights the positive role of “PIL” as a driver of capability. The observed stepwise gradient across PIL levels underscores SH as a proactive strategy to enhance the autonomy and quality of life among older adults. Third, our findings contribute to the diversification of gerontological literature, which has been predominantly Western-centric [45]. By highlighting cultural factors such as “Tham Boon” (merit-making) and the strong community-based support system (for example, Village Health Volunteers), this study underscores the importance of cultural capital. It suggests that non-material resources, social connections and spiritual beliefs are critical assets for successful aging in the Thai context [46, 47].

However, this study has several limitations that should be acknowledged. First, the cross-sectional design precludes causal inferences. Second, a potential selection bias exists. The eligibility criteria required membership in an elderly club. Therefore, the study sample may represent a more socially active and healthier segment of the older population compared to homebound non-members. This phenomenon, often observed in gerontological studies, implies that our results might overestimate the general population’s well-being scores [48]. Additionally, some subgroup sample sizes (those with poor subjective health or specific religious types) were small, which may affect the statistical power and stability of the regression estimates.” Third, the study was conducted in a specific province (Ratchaburi). Cultural nuances regarding “Tham Boon” or family structures may differ in urban Bangkok or other rural regions. Thus, generalizability to the entire Thai population requires caution. Finally, the reliance on self-administered questionnaires may have introduced response bias, such as social desirability bias, particularly in questions regarding religious or family values.

## Conclusion

In conclusion, this study investigated the current states of capability well-being and its association with SH among independent older adults in Central Thailand. The findings revealed that PIL was the strongest independent predictor of the ICECAP-O tariff scores. Better subjective health, perceived financial security, and engagement in caregiving roles were also significantly associated with higher capability well-being. The results suggest that improving well-being in later life requires not only physical and economic support but also focused attention on spiritual and psychosocial dimensions. In the Thai context, where merit-making (Tham Boon) and community-based networks like Village Health Volunteers are fundamental, fostering spiritual meaning and social connectedness plays a critical role. Future research should explore culturally sensitive strategies and interventions that support PIL and social roles to enhance healthy aging in rapidly transitioning super-aged societies.

## Supplementary Information

Supplementary Information 1.

Supplementary Information 2.

Supplementary Information 3.

## Data Availability

Data are available from the authors upon reasonable request.

## Abbreviations

WHO: World Health Organization
SH: Spiritual Health
PIL: Purpose in Life
ICECAP-O: ICEpop CAPability measure for Older people
