# Supplementary material for: Purpose in life as a predictor of capability well-being among older adults in Thailand: a cross-sectional study
Source: BMC Geriatr. 2026 Jul 17;26:1299. doi: 10.1186/s12877-026-07929-2 (PMC13673643; doi:10.1186/s12877-026-07929-2)
Supplement: Supplementary file 1 [file 12877_2026_7929_MOESM1_ESM.docx]

**Supplementary Information 1**

**1. Preparation and Processing for calculating of ICECAP-O tariffs score.**
　The data preparation process followed eight specific steps prior to the primary analysis.
Step 1: All collected raw data including missing values were tabulated in Microsoft Excel.
Step 2: Multiple Imputation (MI) was executed in Stata to address missing data.
Step 3: A single representative dataset was randomly extracted from the 20 imputed datasets generated during the MI process.
Step 4: Data labeling and numerical rounding were performed on the extracted dataset within Stata.
Step 5: The processed dataset was exported back to Excel for ICECAP-O tariffs score calculation.
Step 6: ICECAP-O tariffs score were calculated in Excel according to the model and weight coefficients established by Coast et al. [17] (Supplementary Table 1).
Step 7: The dataset containing the calculated ICECAP-O scores was re-imported into Stata.
Step 8: The finalized dataset was used for all primary statistical analyses within Stata.

**Supplementary Table 1. Attribute of ICECAP-O tariffs score value by Coast et al.**

| **Attribute** | **Value** |
| --- | --- |
| **Attachment** |  |
| I can have all of the love and friendship that I want | 0.2535 |
| I can have a lot of the love and friendship that I want | 0.2325 |
| I can have a little of the love and friendship that I want | 0.134 |
| I cannot have any of the love and friendship that I want | -0.0128 |
| **Security** |  |
| I can think about the future without any concern | 0.1788 |
| I can think about the future with only a little concern | 0.1071 |
| I can only think about the future with some concern | 0.0661 |
| I can only think about the future with a lot of concern | 0.0321 |
| **Role** |  |
| I am able to do all of the things that make me feel valued | 0.1923 |
| I am able to do many of the things that make me feel valued | 0.1793 |
| I am able to do a few of the things that make me feel valued | 0.1296 |
| I am unable to do any of the things that make me feel valued | 0.0151 |
| **Enjoyment** |  |
| I can have all of the enjoyment and pleasure that I want | 0.166 |
| I can have a lot of the enjoyment and pleasure that I want | 0.1643 |
| I can have a little of the enjoyment and pleasure that I want | 0.1185 |
| I cannot have any of the enjoyment and pleasure that I want | 0.0168 |
| **Control** |  |
| I am able to be completely independent | 0.2094 |
| I am able to be independent in many things | 0.1848 |
| I am able to be independent in a few things | 0.1076 |
| I am unable to be at all independent | -0.0512 |
