# Supplementary material for: Purpose in life as a predictor of capability well-being among older adults in Thailand: a cross-sectional study
Source: BMC Geriatr. 2026 Jul 17;26:1299. doi: 10.1186/s12877-026-07929-2 (PMC13673643; doi:10.1186/s12877-026-07929-2)
Supplement: Supplementary file 2 [file 12877_2026_7929_MOESM2_ESM.docx]

**Supplementary Information 2: Questionnaire in English**

**ID No:**

**Health and life questionnaire surveys in Thailand**

**Part 1 Q.1 ~ Q.18: sociodemographic, economic factors, social engagement and health related items**

1． Please tell us your gender. (Please choose one)

　　(1) Male

　　(2) Women

　　(3) Other

2． How old are you in full age?
   Age (	)
3． What kind of school did you last graduate from (or drop out of)? Please choose only one from the following . (Please circle one.)
   (1) Elementary and junior high schools
   (2) High school
   (3) Junior colleges, technical colleges, and vocational schools
   (4) University
   (5) Graduate School
   (6) Other
4． Are you currently married? Please answer only one of the following, including common-law marriages. (Please circle one.)
   (1) Never been married
   (2) Currently have a spouse or partner
   (3) You are divorced from your spouse or partner.
   (4) Your spouse or partner is deceased.
   (5) Other
5． Do you currently have any children? Please answer only one of the following questions, including whether they live together or separately, and whether they are adopted. (Please circle one.)
   (1) With children (living together only)
   (2) With children (living separately only)
   (3) Having children (both living together and separately)
   (4) No children
   (5) Other
6． Who among the following people are currently living with you? Please choose as many as apply from the following, including those who live in a two-family home or in separate buildings on the same property. (You can circle as many as you like.)
   (1) Living alone　　　　　　　　　　　　　　(5) Spouse of child
   (2) Spouse (husband or wife)　　　　　　　　　(6) Grandchild
   (3) Your or your spouse’s parents　　　　　　 (7) Siblings
   (4) Children　　　　　　　　　　　　　　　 (8) Other (	)
7． Are you currently suffering from any illness?
   0. No
   1. Yes (please fill in all that apply)
   (1) Cancer (location: )
   (2) Hypertension
   (3) Diabetes
   (4) Stroke (cerebral hemorrhage, cerebral infarction)
   (5) Cardiovascular diseases (angina pectoris, myocardial infarction, arrhythmia, heart failure, etc.)
   (6) Respiratory diseases (bronchitis, chronic obstructive pulmonary disease (COPD), asthma, etc.)
   (7) Digestive system diseases (gastroduodenal ulcers, etc.)
   (8) Musculoskeletal diseases (osteoporosis, gout, arthropathy, lower back pain, etc.)
   (9) Mental illness (depression, bipolar disorder, schizophrenia, etc.)
   (10) Dementia
   (11) Other (	)
8． Are you currently working and receiving income? Please answer only one of the following. (Please choose one only.)
   (1) Self-employed agriculture, forestry and fishing businesses (including family employees)
   (2) Self-employed commercial or service industry (including family employees)
   (3) Officers of a company or organization
   (4) Full-time employees
   (5) Part-time and temporary employees
   (6) I am not working in a paid job.
   (7)Other
9． What kind of job was the longest you have ever worked in? Please answer only one of the following . (Please circle one.)
   (1) Self-employed agriculture, forestry and fishing businesses (including family employees)
   (2) Self-employed commercial or service industry (including family employees)
   (3) Officers of a company or organization
   (4) Full-time employees
   (5) Part-time and temporary employees
   (6) I am not working in a paid job.
   (7)Other
10． How do you feel about your current financial situation? Please choose only one answer from the following. (Please circle one.)
    (1) My household finances are comfortable and I live without any worries.
    (2) We don't have much financial leeway, but we're living without too many worries.
    (3) My household finances are tight and I am a little worried.
    (4) My household finances are tight and I am very worried.
11． Current household debt situation (Please select only one of the following)
    (1) No debt at all
    (2) I have a small amount of debt that I can manage
    (3) I have a moderate amount of debt and need a repayment plan
    (4) I have a large amount of debt that is a heavy burden
12． If you add up all the income (including pensions) of the household you live with, what is the average amount per month, including tax (including bonuses, if any) ? Please answer by choosing only one of the following . (Please circle one)
    (1) Less than 20,000 bart
    (2) 20,000 bart or more but less than 40,000 bart
    (3) 40,000 bart or more but less than 60,000 bart
    (4) 60,000 bart or more but less than 80,000 bart
    (5) 80,000 bart or more
13． Do you receive a pension ?
    (1) Yes 　(2) No
14． Do you live with someone who needs your care ?
    (1) Yes 　(2) No
15． Do you have faith or devotion? Please choose only one answer from the following. (Please circle one .)
    (1) Very strongly
    (2) Somewhat
    (3) I don't have one
    (4) I don’t know
16． If you answered "(1) Very strongly" or "(2) Somewhat" to the above question, what kind of faith or belief do you have? Please select as many of the following as apply to you. (You can circle as many as you like.)
    (1) Buddhism (5) Taoism
    (2) Shinto (6) Folk beliefs
    (3) Christianity (7) Islam
    (4) Catholicism (8) Other (	)
17． Have you participated in any social activities during the past year that were organized independently by yourself , with friends, or in a group or organization? Please answer as many as you like from the following. (Circle as many as you like.)
    (1) Health and sports (gymnastics, walking clubs, gateball , tai chi, dancing, etc.)
    (2) Hobbies (Haiku, Shigin, Karaoke, Mahjong, Pottery , Go, etc.)
    (3) Local events (taking care of local events such as festivals, etc.)
    (4) Improving living conditions (environmental beautification, greening promotion, urban development, etc.)
    (5) Production and employment (horticulture and animal husbandry for a living, silver human resources centers, etc.)
    (6) Safety management (traffic safety, crime prevention, disaster prevention)
    (7) Educational and cultural awareness activities (study groups, fostering children's groups, transmitting local performing arts, etc.)
    (8) Other
    (9) I have not participated
18． Have you participated in any volunteer activities conducted independently by yourself, with friends, or in a group or organization during the past year? Please answer as many as you like from the following. (Circle as many as you like.)
    (1) Support for the elderly (household assistance, transportation, etc.)
    (2) Childcare support (help with childcare, etc.)
    (3) Other
    (4) I have not participated.
19． How do you feel about your health in general? Please circle the number that applies to you from the following four levels. (Only one circle is allowed.)
    (1) Very healthy
    (2) Fair health
    (3) Not very healthy
    (4) I'm not healthy

**Part 2 Q.20.1 ~ Q.20.5: ICECAP-O (the ICEpop CAPability measure for older people)**

20. Questions about your daily life. Please choose one statement from (1) to (4) for each item that best describes your current daily life and circle it .

20.1 Love and friendship

(1) I can fully experience love and friendship.

(2) I feel a lot of love and friendship.

(3) I can feel a little love and friendship.

(4) I feel no love or friendship at all.

20.2 Concerns about the future

(1) I can think about the future without any particular anxiety.

(2) I can think about the future without much anxiety.

(3) When I think about the future, I feel a little anxious.

(4) When I think about the future, I get very anxious.

20.3 Value

(1) I can do anything that makes me feel worthy.

(2) I can do many things that make me feel valued.

(3) I can do some things that make me feel my own value.

(4) I am unable to do anything that would make me feel worthy of my own actions.

20.4 Pleasure and Joy

(1) I can fully experience pleasure and joy.

(2) I experience a lot of fun and joy.

(3) I can feel some joy and pleasure.

(4) I am unable to experience any pleasure or joy.

20.5 Independence

(1) I am independent in all things.

(2) I am independent in many ways.

(3) I am somewhat independent.

(4) I am not independent at all.

**Part 3 Q.21.1 ~ Q.21.20: the PIL test (the Purpose in Life test)**

21. For each of the following statements, please circle the number that best describes you. (Please try not to choose "4 = neither" as much as possible. )

21.1 I am usually:

completely bored	neither 　　 exuberant, enthusiastic

　　　　　　　　　　　　　1　 2	3	4	5	6	7

└──┴──┴──┴──┴──┴──┘

21.2 Life to me seems:

completely routine	neither	always exciting

　　　　　　　　　　　　　1　 2	3	4	5	6	7

└──┴──┴──┴──┴──┴──┘

21.3 In life, I have:

no goals or desires	neither	very clear goals & desires

　　　　　　　　　　　　　1　 2	3	4	5	6	7

└──┴──┴──┴──┴──┴──┘

21.4 My personal existence is:

　　　　meaningless, without purpose	neither 　 purposeful. meaningful

　　　　　　　　　　　　　1　 2	3	4	5	6	7

└──┴──┴──┴──┴──┴──┘

21.5 Every day is:

exactly the same	neither	constantly new & different

1	2	3	4	5	6	7

└──┴──┴──┴──┴──┴──┘

21.6 If I could choose, I would:

prefer never to have been born	neither	like nine more lives just like this one

　　　　　　　　　　　　　1　 2	3	4	5	6	7

└──┴──┴──┴──┴──┴──┘

21.7 After retiring, I would:

loaf around completely	neither	do exciting things I’ve always wanted to do

　　　　　　　　　　　　　1　 2	3	4	5	6	7

└──┴──┴──┴──┴──┴──┘

21.8 In achieving life goals, I’ve:

made no progress	neither	progressed to complete fulfillment

　　　　　　　　　　　　　1　 2	3	4	5	6	7

└──┴──┴──┴──┴──┴──┘

21.9 My life is:

empty, filled with despair	neither	running over with exciting good things

　　　　　　　　　　　　　1　 2	3	4	5	6	7

└──┴──┴──┴──┴──┴──┘

21.10 If I should die today, I would feel that my life has been:

completely worthless	neither 　　　 very worthwhile

　　　　　　　　　　　　　1　 2	3	4	5	6	7

└──┴──┴──┴──┴──┴──┘

21.11 In thinking of my life, I:

　　 often wonder why I exist	neither　 always see a reason for being here

　　　　　　　　　　　　　1　 2	3	4	5	6	7

└──┴──┴──┴──┴──┴──┘

21.12 As I view the world in relation to my life, the world:

completely confuses me	neither	fits meaningfully with my life

　　　　　　　　　　　　　1　 2	3	4	5	6	7

└──┴──┴──┴──┴──┴──┘

21.13 I am a:

very irresponsible person	neither	very responsible person

　　　　　　　　　　　　　1　 2	3	4	5	6	7

└──┴──┴──┴──┴──┴──┘

21.14 Concerning one’s freedom to make their own choices, I believe a person is:

completely bound by limitations	neither	absolutely free to make all life’s choices

　　　　　　　　　　　　　1　 2	3	4	5	6	7

└──┴──┴──┴──┴──┴──┘

21.15 With regard to death, I am:

unprepared & scared	neither	prepared & unafraid

　　　　　　　　　　　　　1　 2	3	4	5	6	7

└──┴──┴──┴──┴──┴──┘

21.16 With regard to suicide, I have:

thought of it seriously as a way out	neither	never given it a second thought

　　　　　　　　　　　　　1　 2	3	4	5	6	7

└──┴──┴──┴──┴──┴──┘

21.17 I regard my ability to find a meaning, purpose or mission in life as:

practically none	neither	very great

　　　　　　　　　　　　　1　 2	3	4	5	6	7

└──┴──┴──┴──┴──┴──┘

21.18 My life is:

out of my control	neither	in my control

　　　　　　　　　　　　　1　 2	3	4	5	6	7

└──┴──┴──┴──┴──┴──┘

21.19 Facing my daily tasks is:

a painful & boring experience	neither	a source of pleasure/satisfaction

　　　　　　　　　　　　　1　 2	3	4	5	6	7

└──┴──┴──┴──┴──┴──┘

21.20 I have discovered:

no mission or purpose in life	neither	clear‐cut goals and a purpose in life

　　　　　　　　　　　　　1　 2	3	4	5	6	7

└──┴──┴──┴──┴──┴──┘

Thank you for your cooperation.
