# Supplementary material for: Purpose in life as a predictor of capability well-being among older adults in Thailand: a cross-sectional study
Source: BMC Geriatr. 2026 Jul 17;26:1299. doi: 10.1186/s12877-026-07929-2 (PMC13673643; doi:10.1186/s12877-026-07929-2)
Supplement: Supplementary file 3 [file 12877_2026_7929_MOESM3_ESM.docx]

**Supplementary Information 3**

**3. Justification for the Analytical Approach (Why chose mean of ICECAP-O tariffs for analysis):**
　Although the ICECAP-O tariffs score is bounded from 0.0 to 1.0 and its distribution was negatively skewed, we treated it as a continuous variable and employed OLS regression for the following reasons. First, given our sufficiently large sample size (N = 471), the Central Limit Theorem ensures the robustness of OLS estimates against violations of the normality assumption [49]. Second, from a health economics perspective, mean-based estimates are preferred because they provide direct information on the magnitude of effect (marginal effect), which is essential for policy implications [50]. This analytical approach is consistent with previous studies examining determinants of ICECAP-O scores [51, 52, 53]. Additionally, methodological research in the economics of happiness has demonstrated that assuming cardinality for ordinal well-being measures yields estimates substantively equivalent to those derived from ordinal models [54].

　Therefore, we prioritized mean-based analyses to ensure our findings are comparable with existing literature and directly applicable to discussions regarding the magnitude of disparities in capability well-being among older adults.

　Furthermore, univariate analyses for each ICECAP-O domain were conducted to verify the robustness of the multivariate model selection (Supplementary Table 2). As the results confirmed that there was no substantial bias within any specific domain, we employed the mean ICECAP-O tariffs score as the main outcome for the multivariate analysis.

　We primarily used multivariate linear regression analysis. However, 16.4% of the observations were right-censored at the maximum score of 1.0, which is considered moderate censoring and indicates a ceiling effect. As a sensitivity analysis, we also performed Tobit regression to confirm the robustness of our main findings alongside this censoring. The results of the sensitivity analysis using Tobit regression, which calculated to the 16.4% right-censoring. The results were consistent with those of the main multivariate linear regression analysis.

**Reference**

[49] Lumley, T., Diehr, P., Emerson, S., Chen, L. (2002). "The Importance of the Normality Assumption in Large Public Health Data Sets." Annual Review of Public Health, 23, 151–169. DOI: 10.1146/annurev.publhealth.23.100901.140546. <https://doi.org/10.1146/annurev.publhealth.23.100901.140546>
[50] Mihaylova, B., Briggs, A., O'Hagan, A., & Thompson, S. G. (2011). "Review of statistical methods for analysing healthcare resources and costs." Health Economics, 20(8), 897–916. DOI: 10.1002/hec.1653 <https://doi.org/10.1002/hec.165>
[51] Makai, P., Koopmanschap, M. A., Brouwer, W. B. F., & Nieboer, A. P. (2013). "A validation of the ICECAP-O in a population of post-hospitalized older people in the Netherlands." Health and Quality of Life Outcomes, 11, 57. <https://link.springer.com/article/10.1186/1477-7525-11-57>
[52] Couzner, L., Ratcliffe, J., Lester, L., Flynn, T., & Crotty, M. (2013). "Measuring and valuing quality of life for public health research: application of the ICECAP-O capability index in the Australian general population." International Journal of Public Health, 58(3), 367–376. DOI 10.1007/s00038-012-0407-4 <https://link.springer.com/article/10.1007/s00038-012-0407-4>
[53] Baji, P., Farkas, M., Dobos, Á., Zrubka, Z., Kovács, L., Gulácsi, L., & Péntek, M. (2021). "Comparing the measurement properties of the ICECAP-A and ICECAP-O instruments in ages 50–70: a cross-sectional study on a representative sample of the Hungarian general population." The European Journal of Health Economics, 22, 1453–1466. <https://doi.org/10.1007/s10198-021-01325-w>
[54] Ferrer-i-Carbonell, A., & Frijters, P. (2004). "How Important is Methodology for the estimates of the determinants of Happiness?" The Economic Journal, 114(497), 641–659. <https://doi.org/10.1111/j.1468-0297.2004.00235.x>

**Supplementary Table 2 Univariate analysis of each ICECAP-O domain**

| **Modifying Factors** | **ICECAP-O Attachment** | **ICECAP-O Security** | **ICECAP-O Role** | **ICECAP-O Enjoyment** | **ICECAP-O**  **Control** | **ICECAP-O tariffs** |
| --- | --- | --- | --- | --- | --- | --- |
|  | **Mean** | **Mean** | **Mean** | **Mean** | **Mean** | **Mean** |
| **Gender (n = 470*)** | **-** | **-** | **-** | **-** | **-** | **-** |
| Male | 0.223 | 0.126 | 0.170 | 0.157 | 0.179 | 0.855 |
| Female | 0.221 | 0.120 | 0.172 | 0.156 | 0.177 | 0.848 |
| **Age** | ****** | ****** | ****** | ****** | ****** | ** |
| 60-69 | 0.227 | 0.126 | 0.175 | 0.159 | 0.186 | 0.874 |
| 70-79 | 0.223 | 0.123 | 0.171 | 0.157 | 0.173 | 0.846 |
| 80+ | 0.196 | 0.103 | 0.155 | 0.144 | 0.153 | 0.751 |
| **Education Level** | ****** | ****** | ***** | **-** | ****** | ** |
| Primary or lower secondary | 0.216 | 0.117 | 0.169 | 0.155 | 0.174 | 0.833 |
| Upper secondary education / Higher Vocational | 0.231 | 0.128 | 0.178 | 0.161 | 0.183 | 0.881 |
| Bachelor's or Higher | 0.240 | 0.143 | 0.175 | 0.158 | 0.192 | 0.908 |
| **Marital Status** | ****** | ***** | **-** | **-** | ***** | ** |
| Single (never married) | 0.227 | 0.126 | 0.176 | 0.156 | 0.185 | 0.869 |
| Married / Cohabiting | 0.226 | 0.126 | 0.173 | 0.157 | 0.180 | 0.862 |
| Divorced / Living Separately | 0.199 | 0.106 | 0.168 | 0.154 | 0.178 | 0.806 |
| Widowed | 0.220 | 0.120 | 0.167 | 0.157 | 0.168 | 0.831 |
| **Children** | **-** | **-** | **-** | **-** | **-** | - |
| Has children | 0.222 | 0.122 | 0.171 | 0.157 | 0.177 | 0.849 |
| No children | 0.221 | 0.127 | 0.173 | 0.154 | 0.184 | 0.859 |
| **Co-residency Status** | **-** | **-** | **-** | **-** | **-** | - |
| Lives alone | 0.227 | 0.133 | 0.170 | 0.156 | 0.177 | 0.864 |
| Lives with others | 0.221 | 0.121 | 0.172 | 0.157 | 0.178 | 0.850 |
| **Current Illness** | **-** | ***** | ***** | **-** | ****** | * |
| No illness | 0.223 | 0.130 | 0.177 | 0.160 | 0.186 | 0.877 |
| Has illness | 0.221 | 0.119 | 0.169 | 0.155 | 0.174 | 0.840 |
| **Employment Status** | ****** | ***** | ****** | ****** | ****** | ** |
| Worker | 0.230 | 0.126 | 0.175 | 0.159 | 0.184 | 0.874 |
| Not working | 0.207 | 0.116 | 0.165 | 0.153 | 0.166 | 0.806 |
| **Longest Job** | **-** | **-** | **-** | **-** | **-** | - |
| Self-employed | 0.225 | 0.125 | 0.174 | 0.156 | 0.180 | 0.860 |
| Others | 0.221 | 0.121 | 0.171 | 0.157 | 0.177 | 0.847 |
| **Economic Status** | ****** | ****** | ****** | ****** | ****** | ** |
| No difficulty | 0.226 | 0.139 | 0.178 | 0.160 | 0.188 | 0.890 |
| Somewhat manageable | 0.227 | 0.120 | 0.172 | 0.158 | 0.173 | 0.849 |
| Slightly worried | 0.217 | 0.103 | 0.169 | 0.155 | 0.179 | 0.822 |
| Very worried | 0.194 | 0.077 | 0.139 | 0.132 | 0.129 | 0.671 |
| **Household Debt Status** | **-** | ****** | ***** | **-** | **-** | - |
| Has debt | 0.225 | 0.116 | 0.169 | 0.156 | 0.177 | 0.843 |
| No debt at all | 0.217 | 0.133 | 0.176 | 0.158 | 0.180 | 0.864 |
| **Income Level** | ****** | ****** | ****** | ****** | ****** | ** |
| < 20,000 baht | 0.216 | 0.114 | 0.167 | 0.153 | 0.172 | 0.822 |
| 20,000–39,999 baht | 0.231 | 0.133 | 0.180 | 0.163 | 0.186 | 0.892 |
| 40,000+ baht | 0.224 | 0.129 | 0.169 | 0.156 | 0.182 | 0.860 |
| **Pension Receipt Status** | ***** | ****** | ***** | ***** | ***** | ** |
| No | 0.220 | 0.119 | 0.170 | 0.156 | 0.176 | 0.840 |
| Yes | 0.235 | 0.144 | 0.180 | 0.162 | 0.188 | 0.908 |
| **Living with someone who requires your caregiving** | ****** | **-** | **-** | ****** | ****** | ** |
| No | 0.214 | 0.119 | 0.169 | 0.151 | 0.170 | 0.823 |
| Yes | 0.227 | 0.125 | 0.173 | 0.160 | 0.183 | 0.868 |
| **Belief or Faith Status** | ****** | ****** | ****** | ****** | ****** | ** |
| Have and depend on a lot | 0.227 | 0.134 | 0.178 | 0.161 | 0.186 | 0.886 |
| Have and depend on somewhat | 0.222 | 0.118 | 0.171 | 0.157 | 0.176 | 0.843 |
| Do not have or Not sure | 0.198 | 0.105 | 0.149 | 0.138 | 0.162 | 0.751 |
| **Religion Type** | **-** | **-** | ****** | **-** | **-** | - |
| Buddhism | 0.222 | 0.123 | 0.171 | 0.157 | 0.178 | 0.851 |
| Christianity (Protestant) | 0.243 | 0.161 | 0.189 | 0.165 | 0.197 | 0.955 |
| Christianity (Roman Catholic) | 0.220 | 0.118 | 0.177 | 0.159 | 0.182 | 0.856 |
| Animism / Folk beliefs | 0.183 | 0.087 | 0.097 | 0.141 | 0.146 | 0.655 |
| None | 0.215 | 0.101 | 0.166 | 0.142 | 0.172 | 0.795 |
| **Social Activities** | ****** | **-** | ****** | ****** | ****** | ** |
| Does not participate | 0.213 | 0.117 | 0.166 | 0.152 | 0.169 | 0.817 |
| Participates | 0.228 | 0.126 | 0.176 | 0.160 | 0.184 | 0.873 |
| **Volunteering** | ****** | **-** | ***** | ****** | ****** | ** |
| Does not participate | 0.215 | 0.121 | 0.169 | 0.153 | 0.173 | 0.832 |
| Participates | 0.231 | 0.124 | 0.176 | 0.161 | 0.185 | 0.877 |
| **Subjective Health Perception** | ****** | ****** | ****** | ****** | ****** | ** |
| Very good health | 0.246 | 0.158 | 0.188 | 0.165 | 0.201 | 0.958 |
| Good health | 0.230 | 0.128 | 0.176 | 0.160 | 0.187 | 0.882 |
| Not very good health | 0.210 | 0.110 | 0.166 | 0.154 | 0.168 | 0.807 |
| Poor health | 0.160 | 0.082 | 0.120 | 0.110 | 0.078 | 0.551 |
| **PIL** | ****** | ****** | ****** | ****** | ****** | ** |
| Low | 0.180 | 0.087 | 0.141 | 0.135 | 0.136 | 0.678 |
| Moderate | 0.216 | 0.111 | 0.167 | 0.155 | 0.171 | 0.822 |
| High | 0.233 | 0.136 | 0.180 | 0.162 | 0.190 | 0.901 |

*・Indicating univariate significance: * p-value <0.05, ** p-value<0.01
・Continuous outcomes(ICECAP-O tariffs) were compared using the t-test (two groups) or One-way ANOVA (≧ 3 groups).*
